# Case Report: Donor-derived herpes simplex virus type 1 hepatitis in a kidney transplant recipient with fatal outcome

**DOI:** 10.3389/frtra.2025.1591855

**Published:** 2025-05-13

**Authors:** Jørn Petter Lindahl, Christina Dörje, Grete Birkeland Kro, Krzyztof Grzyb, Harald Hugenschmidt, Johannes Espolin Roksund Hov, Andreas Barratt-Due, Karsten Midtvedt

**Affiliations:** ^1^ Section of Nephrology, Department of Transplant Medicine, Oslo University Hospital, Rikshospitalet, Oslo, Norway; ^2^ Department of Microbiology, Oslo University Hospital, Rikshospitalet, Oslo, Norway; ^3^ Department of Pathology, Oslo University Hospital, Rikshospitalet, Oslo, Norway; ^4^ Section of Transplant Surgery, Department of Transplant Medicine, Oslo University Hospital, Rikshospitalet, Oslo, Norway; ^5^ Section of Gastroenterology, Department of Transplant Medicine, Oslo University Hospital, Rikshospitalet, Oslo, Norway; ^6^ Department of Anesthesia and Intensive Care Medicine, Division of Emergencies and Critical Care, Oslo University Hospital, Rikshospitalet, Oslo, Norway

**Keywords:** donor-derived infection, herpes simplex virus, kidney transplantation, immunosuppression, hepatitis, case report

## Abstract

Current screening practices have significantly reduced the transmission of donor-derived infections through organ transplantations. However, in exceptional cases, a deceased donor may harbor an undetected active infection, or abnormal blood test results may be mistakenly attributed to the dying process, resulting in missed infections. These ongoing infections can then be transmitted through the grafts. This report presents a case of confirmed donor-derived herpes simplex virus type 1 (HSV-1) hepatitis following kidney transplantation. The HSV-1 infection in the recipient was initially overlooked and misattributed to a probable mycophenolate mofetil-induced etiology, which led to a delay in initiating antiviral therapy. The recipient subsequently developed HSV-1 hepatitis, which progressed to liver failure and multiorgan failure, ultimately resulting in death. As a result of this case, our transplant center promptly revised its screening and prophylactic antiviral treatment protocols. All kidney transplant recipients who are herpes simplex virus (HSV) antibody-negative now receive valaciclovir until the donor's HSV DNA PCR status is confirmed to be negative.

## Introduction

Recipients of solid organ transplants are at risk of donor-derived transmission of various pathogens, including viruses, bacteria, fungi, and protozoa (1–3). Some viruses, including herpes simplex virus (HSV), are particularly prone to reactivation during immunosuppressive therapy, which can lead to adverse outcomes after solid organ transplantation (SOT). The clinical outcome for the recipient may depend on whether the infection is a reactivation of a latent virus or a primary infection. Therefore, the pre-transplant serological screening of donors and recipients is recommended to mitigate such risks (4). Certain donor-derived infections can be anticipated, such as when organs from a cytomegalovirus (CMV)-seropositive donor are transplanted into a CMV-seronegative recipient, in which case prophylactic antiviral treatment is routinely prescribed for 3–6 months (4, 5).

Primary HSV infections can present asymptomatically or with localized lesions, such as those affecting the skin, mouth, or genital areas. Following primary infection, HSV typically becomes dormant in sensory neurons and is reactivated intermittently, usually presenting as localized recurrent lesions (6, 7). In rare cases, HSV can disseminate and affect other organs, resulting in meningitis, encephalitis, hepatitis, or pneumonitis (6–8). Reactivation of latent HSV can occur either locally or systemically, and immunosuppressed patients are at greater risk of reactivation. Additionally, HSV viremia has previously been reported in up to 30% of immunocompetent critically ill patients, with the incidence increasing with prolonged stay in the intensive care unit (ICU) (9, 10).

Approximately two-thirds of transplant recipients are seropositive for HSV at the time of transplantation, leaving one-third at risk of primary HSV infection (11, 12). Consequently, most symptomatic HSV-related diseases in adult transplant recipients are caused by viral reactivation. However, in many countries, HSV serology is not routinely conducted in transplant settings, despite the potential for severe disease in solid organ transplant recipients (13, 14). Primary donor-derived infections with herpes simplex type 1 or 2 (HSV-1/2) have been reported in liver and kidney transplant recipients, and these infections can be particularly severe in the early post-transplant period owing to the absence of immunogenic memory (15–18).

While routine HSV screening for serological status was conducted at our national transplant center, no specific clinical actions have been previously outlined when a kidney from an HSV-seropositive donor is transplanted into an HSV-seronegative recipient. Here, we present a case of donor-derived primary HSV-1 infection following kidney transplantation, which resulted in a fatal outcome.

## Case presentation

A 59-year-old male with end-stage renal disease, most likely secondary to hypertension and type 2 diabetes mellitus. A biopsy was not performed because the patient had only one functioning kidney and the other was atrophic. Prior to enlisting, the patient had completed six months of anti-tuberculosis treatment for latent tuberculosis. The patient had been on the waiting list for approximately 30 months and remained pre-dialytic, with an estimated glomerular filtration rate of 15 ml/min/1.73 m² at the time of engraftment.

The patient received a kidney from a brain dead donor. The donor, a 73-year-old male, died of intracerebral hemorrhage, with a creatinine level of 68 µmol/L at the time of donation. The donor-recipient blood group compatibility was B-B, with a human leukocyte antigen mismatch of 1/2/2 (A/B/DR), and the cold ischemia time was 13 h and 5 min. Both the donor and recipient were CMV IgG antibody-positive; in accordance with institutional protocols, valganciclovir prophylaxis was not administered (19). No surgical complications were observed, and the patient's creatinine level decreased on the first postoperative day (Table 1). Immunosuppressive therapy was administered in accordance with the standard protocol for patients with low immunological risk (20). Induction therapy consisted of a single dose of 250 mg methylprednisolone on day 0 and 20 mg intravenous basiliximab on days 0 and 4. Maintenance immunosuppression included tacrolimus with target trough levels of 4–7 ng/ml, mycophenolate mofetil (MMF) 750 mg twice daily (BID), and prednisolone starting at 20 mg/day and tapering to 5 mg daily after six months. Additionally, a six-month course of trimethoprim/sulfamethoxazole (TMS) 80 mg/400 mg was administered for Pneumocystis jirovecii prophylaxis.

**Table 1:** Recipient bloodwork following transplantation.

Variable (reference)	Time after transplantation (days)														
	0 Day of Tx	1	2	3	4	5	6	7	8	9	10	11	12	13	14 Day of death
Hb (13.4–17.0 g/dl)	10.7	8.7	8.2	8.0	7.8	9.9	9.9	10.1	9.0	9.3	8.8	9.1	8.8	7.9	7.3
Platelets (145–390 × 10⁹/L)	123	115	106	95	96	109	106	122	107	94	69	50	37	15	95
Leukocytes (3.5–10.0 × 10⁹/L)	4.6	7.3	9.7	8.9	7.2	7.0	6.2	5.5	3.5	2.5	1.2	0.9	2.1	3.7	4.9
Creatinine (60–105 µmol/L)	340	219	166	166	158	147	129	130	153	155	192	178	161	277^a^	n/a^b^
CRP (<4 mg/L)	<0.6	3.6	2.3	2.0	2.2	1.9	2.2	3.3	12	31	28	20	12	11	6.7
ASAT (15–45 U/L)	28	n/a	n/a	n/a	60	n/a	n/a	n/a	n/a	n/a	n/a	5,451	6,392	12,153	n/a
ALAT (10–70 U/L)	27	n/a	n/a	n/a	63	n/a	n/a	n/a	n/a	n/a	n/a	3,639	3,889	5,884	n/a
Bilirubin (5–25 µmol/L)	4	n/a	n/a	n/a	4	n/a	n/a	n/a	n/a	n/a	n/a	10	18	53	46
INR (0.9–1.2 N-Ratio)	1.0	n/a	n/a	n/a	1.1	n/a	n/a	n/a	1.1	1.2	n/a	1.4	1.4	2.2	3.8
Ferritin (30–400 µg/L)	n/a	n/a	n/a	n/a	n/a	n/a	n/a	n/a	n/a	n/a	n/a	69,673	n/a	194,449	363,067
HSV-1 IgM (±)															
Donor	-														
Recipient	-												-		
HSV-1 IgG (±)															
Donor	+														
Recipient	-											-	-		
HSV-1 DNA PCR (±, Ct^c^)															
Donor	+, 36														
Recipient	-				+, 36							+, 17	+, 16		+, 17

^a^ Start of CRRT.

^b^ n/a, not available.

^c^ Ct, cycle threshold.

The patient's initial postoperative period was uneventful. However, on postoperative day 7, the patient developed low-grade fever (37.8°C) and moderate headache. Repeated PCR testing for SARS-CoV-2 using nasopharyngeal swabs yielded negative results. Chest radiography was inconclusive, and on postoperative day 8, intravenous cefotaxime was initiated because of a suspected bacterial infection, although CRP levels remained only mildly elevated (Table 1). Blood and urine cultures were negative and repeated CMV PCR tests were negative, thereby ruling out CMV reactivation.

By postoperative day 9, the patient's fever persisted at 39.1°C, and his serum creatinine had increased from 130 µmol/L–155 µmol/L. Ultrasonography of the kidney transplant revealed no abnormal findings. Despite the persistent fever and headache, the patient's general condition remained relatively stable. On postoperative day 10, blood tests revealed leukopenia (Table 1), prompting a pause in TMS administration and a reduction in the MMF dose from 750–500 mg BID. Owing to continued deterioration in kidney function (creatinine rising to 192 µmol/L), a kidney transplant biopsy was performed, which did not reveal evidence of acute rejection (Banff classification: i0t1v0, C4d negative) and did not explain the fever. Despite negative biopsy findings, the patient was treated with intravenous methylprednisolone (500 mg + 250 mg).

**Figure 1:**
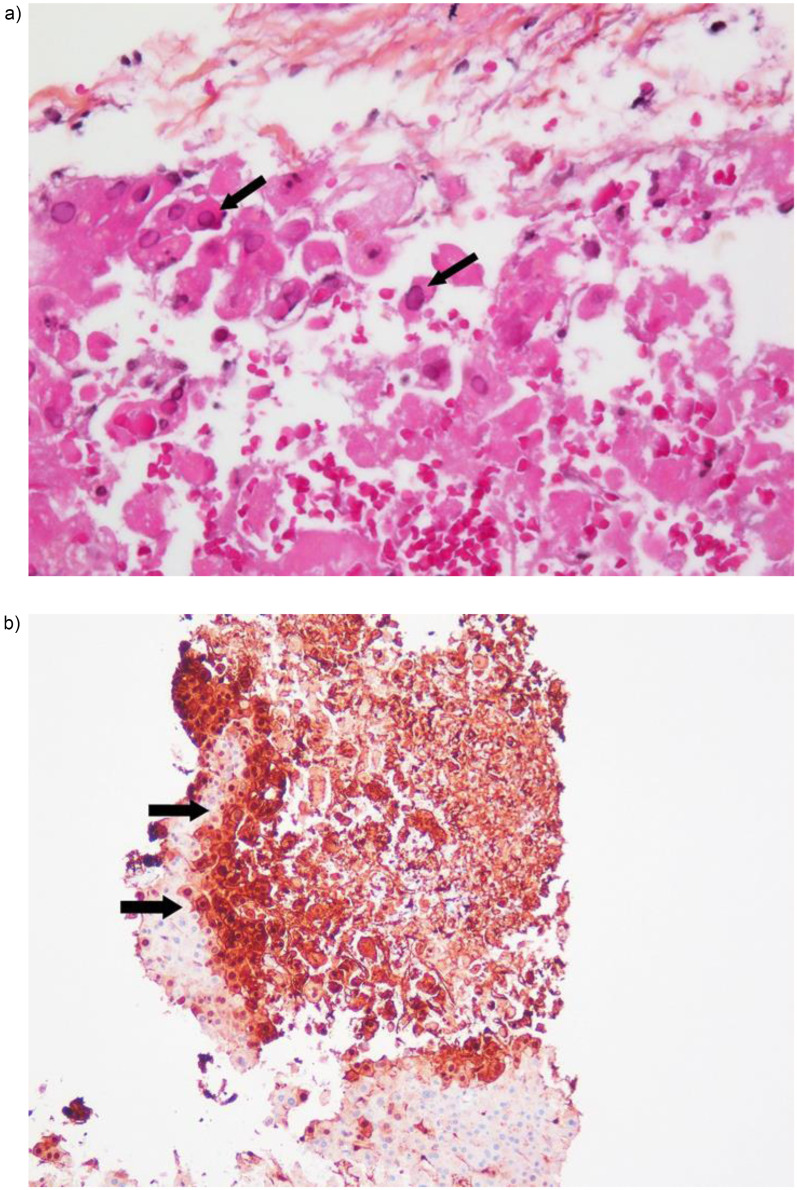
**(a)** Liver biopsy showing necrosis and typical amophilic, glassy intranuclear inclusions in hepatocytes (arrows) (hematoxylin-eosin staining x40). **(b)** Immunohistochemistry revealing abundant herpes virus antigens in areas of necrosis (arrows) (HSV immunostaining of liver biopsy).

On postoperative day 11, the liver enzyme levels showed a significant increase (Table 1). Liver ultrasonography revealed no gross pathological findings. Different diagnostic possibilities were discussed as the cause of the increase in liver transaminase levels, including toxic, circulatory, or viral causes. Given this uncertainty, intravenous aciclovir was initiated to treat the potential viral infections. Subsequent liver biopsy revealed predominantly necrotic liver tissue, consistent with viral etiology, and immunohistochemistry confirmed the diagnosis of HSV hepatitis (Figure 1).

By postoperative day 12, the patient's condition had significantly deteriorated, with an altered mental status and confusion. Blood tests indicated liver failure and disseminated intravascular coagulation, necessitating transfer to the ICU for further management. PCR testing for HSV-1 DNA in the blood was positive, with a cycle threshold (Ct) value of 16 (Table 1). Although quantitative HSV-1 DNA PCR was not feasible, a Ct value of 16 indicated significant viral load. The patient was administered intravenous immunoglobulin (0.4 g/kg), broad-spectrum antibiotics (piperacillin/tazobactam), antivirals (aciclovir), and antifungal (anidulafungin).

Despite these interventions, the patient's condition continued to worsen, requiring intubation and mechanical ventilation, and continuous renal replacement therapy. The tacrolimus dose was reduced from 5 mg BID–3 mg BID, and the MMF was temporarily halted. Consideration was given to acute liver transplantation due to liver failure; however, because of ongoing HSV-1 viremia, as evidenced by a PCR Ct value of 16–17, and the patient's critical condition, liver transplantation was deemed to be contraindicated. The patient ultimately succumbed to acute HSV-1 hepatitis, leading to multiorgan failure on postoperative day 14. Autopsy confirmed the clinical diagnosis.

Retrospective analysis of donor blood revealed that the donor was IgG-positive for HSV-1, IgM-negative, and exhibited low-level active viremia, as evidenced by HSV-1 DNA PCR with a Ct value of 36, suggesting low viremia.

## Discussion

In this case report, we describe a donor-derived primary HSV-1 infection in a kidney transplant recipient who developed HSV-1-induced hepatitis, leading to secondary liver and multiorgan failure, and ultimately died two weeks post-engraftment. To the best of our knowledge, this is the first reported case of fatal donor-derived HSV-1-related hepatitis following kidney transplantation at our center.

During SOT, there is an inherent risk of donor-derived disease transmission to the recipient. Certain donor-derived infections, such as cytomegalovirus (CMV), are well recognized and occur with considerable frequency. Post-transplant management typically involves either prophylactic administration of valganciclovir for a defined period or the implementation of preemptive treatment strategies (4, 5). In contrast, unexpected donor-derived disease transmission is relatively rare, with an estimated incidence of less than 1% among transplant recipients (21). Infectious pathogens represent the most frequently implicated agents in these cases. Notably, a limited number of cases of donor-derived HSV infection resulting in secondary hepatitis and fatal outcomes have been documented in the context of solid organ transplantation (15–18). Fever, leukopenia, and hepatitis are the hallmark signs of disseminated HSV infection. As observed in our patient, severe visceral disease may initially manifest without the accompanying mucocutaneous lesions. Consequently, it is essential that HSV and other viral infections be considered in the differential diagnosis regardless of the recipient's serological status prior to transplantation.

The clinical presentation of drug-induced hepatitis, particularly MMF, closely resembles that of viral hepatitis. However, in solid organ transplant recipients who develop hepatitis followed by multiorgan failure, the underlying cause is often an acute viral infection. Therefore, early diagnostic evaluation, including culture and blood tests, is critical, and treatment should be initiated promptly, often before full diagnostic results are available or conclusive.

In the present case, an increase in transaminase levels was noted on day 4 post-engraftment, but transaminase levels were not adequately managed until day 11. This delay may have contributed to the postponement of accurate diagnosis and timely intervention. In the updated and recently published guidelines for cytomegalovirus (CMV) management in kidney transplant recipients, six months of prophylaxis with valganciclovir (or letermovir) or a preemptive strategy is recommended for CMV-seropositive donor/seronegative recipient (D+/R−) constellations (22). Additionally, for all CMV IgG-positive recipients, three months of valganciclovir preemptive therapy is advised. It is worth noting that valganciclovir prophylaxis confers incidental protection against early herpes zoster virus (HZV) infections (23). At our center, CMV prophylaxis is typically administered only when a CMV IgG-positive donor is transplanted into a CMV IgG-negative recipient. For all other recipient constellations, including the case described here, a preemptive management strategy is employed. Although the risk of early post-transplant HSV infection remains poorly defined, intensified immunosuppression (e.g., methylprednisolone for suspected organ rejection, as in our patient) can exacerbate the risk of HSV recurrence or accelerate the ongoing primary infection. However, to the best of our knowledge, data on the role of HSV prophylaxis during rejection episodes are lacking.

The prevalence of herpes simplex virus type 1 (HSV-1) seropositivity in the adult population is high, and may reach 70%–90% among SOT recipients (11, 12). It is estimated that 5%–10% of SOT recipients develop symptomatic HSV-1 infection following transplantation (24). In the majority of cases, these infections represent reactivations of latent virus and are rarely life-threatening. In the present case, the kidney donor was found to be HSV-1 IgG-positive and IgM-negative at the time of donation, suggesting a past infection with HSV-1. However, retrospective HSV-1 DNA PCR testing revealed active HSV-1 viremia at the time of the donation. Studies have shown that systemic reactivation of HSV-1 is common in critically ill patients, and the risk of HSV-1 viremia in donors from this group is considerable (9, 10).

This unfortunate case prompted revision of the transplant protocol. Specifically, when a deceased donor is anti-HSV antibody-positive and donates a kidney to an HSV antibody-negative recipient, valaciclovir is administered to the recipient until it can be determined via PCR that the donor is negative for HSV DNA. In cases where the donor tests positive for HSV DNA via PCR, valaciclovir therapy is maintained for three months post-transplantation. Although the transmission of infectious agents is rare, delayed detection can, as illustrated in our case, lead to adverse outcomes for recipients. Such events may undermine public confidence in the SOT process and must therefore be prevented. We sincerely hope that other transplant centers will benefit from the lessons learned from our experiences.

## Data Availability

The original contributions presented in the study are included in the article/Supplementary Material, further inquiries can be directed to the corresponding author.
